# Dissecting the Role of the FGF19-FGFR4 Signaling Pathway in Cancer Development and Progression

**DOI:** 10.3389/fcell.2020.00095

**Published:** 2020-02-20

**Authors:** Yanan Liu, Meng Cao, Yuepiao Cai, Xiaokun Li, Chengguang Zhao, Ri Cui

**Affiliations:** ^1^Cancer and Anticancer Drug Research Center, School of Pharmaceutical Sciences, Wenzhou Medical University, Wenzhou, China; ^2^Wenzhou University-Wenzhou Medical University Collaborative Innovation Center of Biomedical, Wenzhou, China; ^3^Institute of Life Sciences, Wenzhou University, Wenzhou, China

**Keywords:** FGF19, FGFR4, inhibitors, cancer, targeted therapy

## Abstract

Fibroblast growth factor (FGF) receptor 4 (FGFR4) belongs to a family of tyrosine kinase receptor. FGFR4 is highly activated in certain types of cancer and its activation is closely associated with its specific ligand, FGF19. Indeed, FGF19-FGFR4 signaling is implicated in many cellular processes including cell proliferation, migration, metabolism, and differentiation. Since active FGF19-FGFR4 signaling acts as an oncogenic pathway in certain types of cancer, the development and therapeutic evaluation of FGFR4-specific inhibitors in cancer patients is a topic of significant interest. In this review, we aim to provide an updated overview of currently-available FGFR4 inhibitors and their ongoing clinical trials, as well as upcoming potential therapeutics. Further, we examined the possibility of enhancing the therapeutic efficiency of FGFR4 inhibitors in cancer patients. We also discussed the underlying molecular mechanisms of oncogenic activation of FGFR4 by FGF19.

## Introduction

The fibroblast growth factor receptors (FGFRs) consist of four highly conserved transmembrane receptor tyrosine kinases (FGFR1-4) (Lin and Desnoyers, 2012). When FGFs bind to their specific FGFRs, they subsequently activate downstream signaling and regulate many biological processes including embryonic development, cell proliferation, differentiation, and tissue repair (Wilkie et al., 1995). FGF-FGFR signaling is dysregulated in many diseases, including cancers. Compared to the other three FGFR family members, the function of the FGFR4 signaling pathway in cancer is less well-characterized. Recently, it has been reported that the activation of FGF19-FGFR4 signaling is closely associated with cancer development and progression (Zhang et al., 2011; Chen et al., 2019), suggesting that FGF19-FGFR4 might be an attractive target for effective anticancer therapeutics (Schadt et al., 2018; Gao et al., 2019).

## Molecular Characteristics of Fgfr4

**FIGURE 1:**
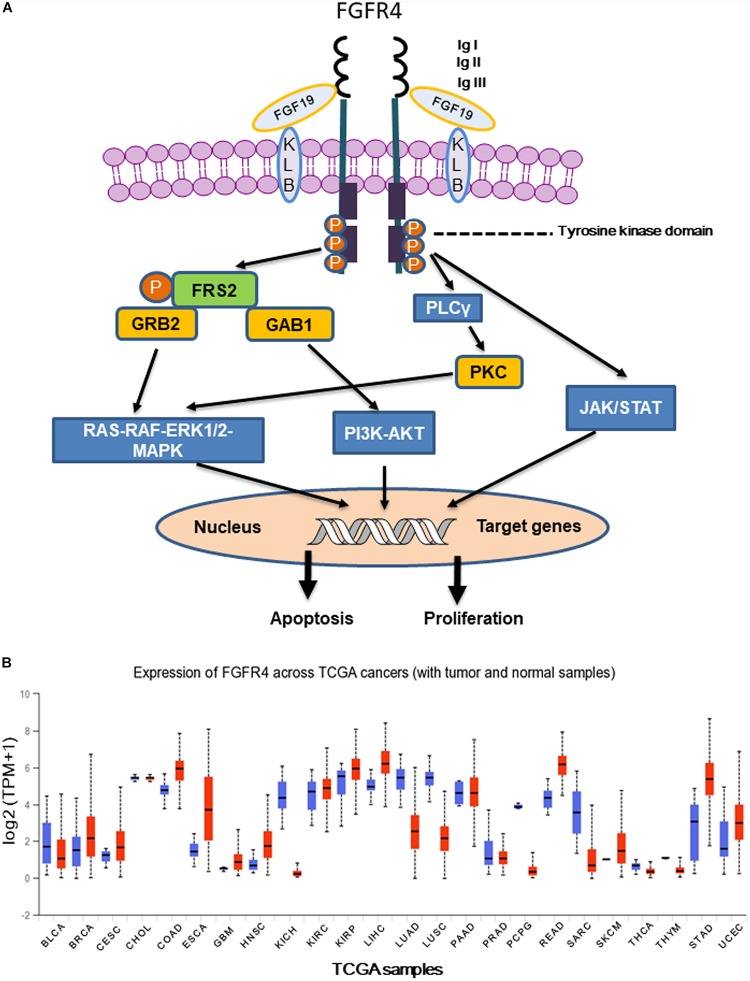
**(A)** Following FGFR4 and Klotho (KLB) bind to FGF19, activated FGFR4 forms homo- or heterodimer and subsequently activates multiple downstream signaling pathways including Ras-Raf- MAPK, PI3K-AKT, EMT, and JAK/STAT. **(B)** Expression of FGFR4 in various The Cancer Genome Atlas (TCGA) cancer types by http://ualcan.path.uab.edu/cgi-bin/Pan-cancer.pl?genenam=FGFR4, X axis represents 33 TCGA different cancer types (ACC, adrenocortical carcinoma; BLCA, bladder urothelial carcinoma; BRCA, breast invasive carcinoma; CESC, cervical squamous cell carcinoma and endocervical adenocarcinoma; CHOL, cholangio carcinoma; COAD, colon adenocarcinoma; DLBC, lymphoid neoplasm diffuse large B-cell lymphoma; ESCA, esophageal carcinoma; GBM, glioblastoma multiforme; HNSC, head and neck squamous cell carcinoma; KICH, kidney chromophobe; KIRC, kidney renal clear cell carcinoma; KIRP, kidney renal papillary cell carcinoma; LAML, acute myeloid leukemia; LGG, brain lower grade glioma; LIHC, liver hepatocellular carcinoma; LUAD, lung adenocarcinoma; LUSC, lung squamous cell carcinoma; MESO, mesothelioma; OV, ovarian serous cystadenocarcinoma; PAAD, pancreatic adenocarcinoma; PCPG, pheochromocytoma and paraganglioma; PRAD, prostate adenocarcinoma; READ, rectum adenocarcinoma; SARC, sarcoma; SKCM, skin cutaneous melanoma; STAD, stomach adenocarcinoma; TGCT, testicular germ cell tumors; THCA, thyroid carcinoma; THYM, thymoma; UCEC, uterine corpus endometrial carcinoma; UCS, uterine carcinosarcoma; UVM, uveal melanoma). Y axis represents log_2_ (TPM + 1) transformed expression data for plotting (TPM, transcripts per million).

FGFR4 is a member of the FGFR family, contains tyrosine kinase domains, and plays a critical role in embryonic development, tissue repair, tumor angiogenesis, and tumor progression (Wu et al., 2011). The human FGFR4 gene is located on the long arm of chromosome 5 (5q35.1) and spans a genomic region of about 11 kb. The *FGFR4* gene has five transcript variants with three of them encoding the FGFR4 isoform 1 with 802 amino acids. The estimated molecular weight of FGFR4 isoform 1 is around 95–110 kDa, depending on its glycosylation levels (Raja et al., 2019). The basic protein domain structure of the FGFR4 is composed of four parts including a signaling peptide, three extracellular immunoglobulin (Ig) domains, a transmembrane domain, and an intracellular tyrosine kinase domain (Figure 1A). Similar with FGFR1-3, the extracellular region of FGFR4 has Ig-like domains (IgI, IgII, and IgIII), which support specific ligand-binding, including FGF19. Unlike FGFR1-3, FGFR4 does not have splice variants in IgIII, which generates the IIIb and IIIc transcript variants encoding different receptor isoforms. Therefore, single ligand binding domain of FGFR4 enables high-affinity bindings of its specific ligands, suggesting that it would be possible to develop highly specific inhibitors (Touat et al., 2015). Genetic alterations of FGFRs were found in 7.1% of cancers. Compared with the other three members of the FGFR family (about 7%), genetic alternations of FGFR4 (0.5%) were relatively infrequent. Gene amplification was most frequent type of alteration in all FGFR4 gene alterations with frequency of 69% (Presta et al., 2017). Overexpression of FGFR4 has been reported in several solid tumors including hepatocellular carcinoma (HCC), breast cancer, oropharyngeal squamous cell carcinoma (OPSCC), and pancreatic cancer (Lang and Teng, 2019). High expression of FGFR4 was reported to be associated with poor overall survival of NSCLC (Huang et al., 2015) and malignant pleural mesothelioma (Vlacic et al., 2019). Particularly, FGFR4 expression was significantly upregulated in most liver cancer cases, and enhanced FGF19-FGFR4 signaling is linked to HCC progression, metastasis, and poor survival (Sawey et al., 2011). Although high expression of FGFR4 was observed in OPSCC and pancreatic cancer, FGFR4 does not show prognostic significance in these cancers (Motoda et al., 2011; Koole et al., 2015).

## Molecular Characteristics of Fgf19

The human FGF family is comprised of 22 members that are divided into seven subfamilies according to their sequences and biochemical functions. FGFs are involved in broad biological processes including embryogenesis, angiogenesis, tissue homeostasis, and cancer progression (Presta et al., 2017). The FGF19 subfamily is called hormone-like FGFs and includes FGF19, FGF21, and FGF23. Unlike other FGF families that bind to heparin-sulfate proteoglycan for paracrine signaling by FGFRs, FGF19 subfamily binds to FGFRs and its co-receptor, Klotho, to transfer endocrine signaling (Katoh, 2016). β-Klotho (KLB) forms a complex with FGFR4, enhancing the affinity between FGF19 and FGFR4 (Repana and Ross, 2015). Promoter hypermethylation induced downregulation of KLB was frequently seen in breast, colon, pancreatic and stomach cancers (Dallol et al., 2015). In contrast, elevated expression of KLB and FGFR4 was reported in HCC (Poh et al., 2012), suggesting dual functions of KLB in cancer development and progression. FGF19 subfamily functions as a secreted endocrine signal that regulates various metabolic process. FGF19 promotes protein synthesis by activating mTOR signaling, and enhances glycogen synthesis through inhibition of GSK3α and GSK3β. In contrast, FGF19 reduces gluconeogenesis by inhibiting cAMP regulatory element binding protein/PGC1A axis (Prieto-Dominguez et al., 2018). The FGF19 genomic locus is on the chromosome 11q13.3, and is frequently amplified in human cancers. For example, amplification of the FGF19 genomic locus including the well-established proto-oncogene CCND1, was found in liver cancer, breast cancer, lung cancer, bladder cancer and esophageal cancer (Sawey et al., 2011; Hoover et al., 2015; Tiong et al., 2016; Zhang et al., 2017). Hyperactivation of FGFR4 by FGF19 was reported in colon cancer cells and hepatocellar carcinoma. An animal model showed that a neutralizing antibody against FGF19 was able to abrogate liver cancer development in transgenic mice and inhibited xenograft tumors from HCC and colon cancer, suggesting feasibility of FGF19 as a therapeutic target (Desnoyers et al., 2008; French et al., 2012). Particularly, the functions of FGF19 are well-characterized in HCC. FGF19 gene expression is significantly upregulated in HCC tissues compared to normal adjacent liver tissues, which suggests that the amplified FGF19 genomic locus might be directly linked to upregulated FGF19 gene expression (Zhao H. et al., 2016). In addition, serum FGF19 levels are significantly higher in preoperative HCC patients than post-operative HCC patients, suggesting that FGF19 may serve as a non-invasive biomarker of disease (Miura et al., 2012). Recently, Hyeon et al. (2013) analyzed 281 patients who underwent curative resection and found that 48% of the HCC tissues had overexpressed FGF19 protein. FGF19 expression was positively associated with a larger tumor size, more advanced Barcelona Clinic Liver Cancer (BCLC) stage, early recurrence, and poor prognosis.

## Downstream Pathways of Fgf19-Fgfr4

Accumulating evidence indicates that FGFR4 plays a critical role in cancer progression and metastasis, particularly in those cancer patients who have FGF19 amplification. Klotho interacts with FGFR4 forming 1:1 heterocomplex to facilitate binding between FGF19 and FGFR4. Later FGF19 binds to heterocomplex of Klotho and FGFR4 to form homodimer receptor complex, activating downstream signaling pathways (Gao et al., 2017; Lang and Teng, 2019; Raja et al., 2019). Here, we summarized and discussed four main signaling pathways downstream of FGF19-FGFR4 signaling including Ras-Raf-MAPK, PI3K-AKT, epithelial–mesenchymal transition (EMT), and the signal transducer and activator of transcription (STAT) pathway (Figure 1A).

## FGFR4-Mediated Activation of Ras/Raf/MAPK and PI3K-AKT Pathways Promotes Tumor Growth

Ras/Raf/MAPK and PI3K-AKT pathways are two major pathways that are frequently dysregulated in cancer. The Ras/Raf/MAPK pathway is aberrantly activated in a number of cancers, and is involved in the regulation of cell proliferation, migration, survival, and apoptosis (Cary et al., 1999; Stacey, 2003; Boonstra et al., 2013). When FGFR4 is activated, phosphorylated FGFR4 recruits and phosphorylates intracellular specific adaptor protein FGFR-substrate-2 (FRS2), which in turn recruits the adaptor proteins GRB2 and SOS, subsequently activating the Ras-Raf-MAPK pathway. Meanwhile, GRB2 binds to GAB1, leading to activation of the PI3K-AKT pathway (Touat et al., 2015). PI3K-AKT signaling leads to decreased apoptosis and increased cell growth/proliferation. High activation of the PI3K-AKT pathway has been noted in a number of cancers including prostate cancer (Toren and Zoubeidi, 2014), lung cancer (Qian et al., 2006), and HCC (Zhou et al., 2011). On the other hand, once activated, FGFR4 phosphorylates phospholipase Cγ (PLCγ) which in turn activates protein kinase C (PKC), and subsequently stimulates the Ras/Raf/MAPK pathway, contributing to cell proliferation and migration (Goetz and Mohammadi, 2013).

## The FGF19-FGFR4 Signaling Pathway Enhances EMT

The FGF19-FGFR4 axis has been reported to promote the metastatic potential of HCC. When activated, the FGF19-FGFR4 pathway enhances GSK3β-βcatenin signaling, consequently inducing EMT and resulting in increased HCC metastasis (Goetz and Mohammadi, 2013; Zhao H. et al., 2016). In addition, activated FGFR4 stimulates AKT, ERK1/2, and Src, promoting the invasive activity of colon cancer (CRC) cells. Inversely, knockdown of FGFR4 reduces the migratory and invasive ability of CRC cells by upregulating epithelial marker E-cadherin and downregulating mesenchymal marker Snail, suggesting a pivotal role of FGFR4 in CRC metastasis (Peláez-García et al., 2013). Moreover, increased activation of the FGFR4-GSK3β-βcatenin axis was also reported in CRC metastasis. FOXC1 was found to directly bind to the promoter region of FGFR4 and enhance FGFR4 expression. High levels of FOXC1 have been reported to be correlated with CRC metastasis, recurrence, and poor prognosis (Liu et al., 2018).

## STATs Signaling Pathway

The JAKs-STATs pathway is an efficient and well-organized system which is principally dedicated to the regulation of gene expression (Zhao C. G. et al., 2016). Basically, activation of JAKs-STATs pathway requires cytokine-mediated phosphorylation and activation of non-receptor tyrosine kinases, JAKs (Nicolas et al., 2013). Once FGF19 binds to FGFR4/Klotho complex, FGF19-dependent FGFR4 dimerization leads to a conformational change in its structure, which facilitates intermolecular transphosphorylation and recruits downstream adaptor proteins for further activation of signaling cascades (Touat et al., 2015). It has been reported that FGF19 acts through the receptor complex FGFR4-KLB to activate JAKs/STATs signaling. FGF19-FGFR4 dimerization induces JAKs phosphorylation and activation, and activated JAKs phosphorylate STATs (Zhou et al., 2014). Activated STATs then form homo- or heterodimers to translocate to the nucleus where they carry out their function as a transcription factor (Nicolas et al., 2013). In addition, a single nucleotide polymorphism, rs351855 (G/A) results in an amino acid change in the FGFR4 protein, producing the Arg388-variant. The FGFR4 Arg388-variant enhances STAT3 signaling, promotes tumor progression, and is associated with poor prognosis of multiple cancers (Ulaganathan et al., 2015; Kogan et al., 2018). In the Rhabdomyosarcoma (RMS), mutations in FGFR4 tyrosine kinase domain (K535 and E550) might activate STAT3 signaling, increasing tumor growth and metastatic potential in mice model (Taylor et al., 2009).

## The FGF19-FGFR4 Axis in Cancer

Increasing evidence indicates that the FGF19-FGFR4 pathway plays a pivotal role in multiprocess of cancer initiation and progression (Wu et al., 2010, 2011). Recombinant FGF19 promotes proliferation and the invasive ability of HCC cells, however, knockdown of FGF19 or FGFR4 by siRNA inhibits proliferation and invasion and induces apoptosis of HCC cells. FGF19 is highly expressed in HCC, and patients with low FGF19 expression have prolonged 5-year survival rates compared to those patients with high FGF19 expression (Miura, 2012). Overexpression of FGF19 in transgenic mice has been shown to promote liver tumor formation by enhancing the expression of α-fetoprotein (AFP), an oncofetal protein used as a marker for neoplastic transformation of hepatocytes (Nicholes et al., 2002). Meanwhile, FGFR4 has been reported to contribute to HCC progression by regulating AFP secretion, proliferation, and preventing apoptosis of HCC cells (Han et al., 2009).

In breast cancer, upregulation of FGFR4 is partly due to gene amplification. Co-expression of FGFR4 and FGF19 was observed in about 30% of primary breast cancers. Furthermore, FGFR4 and FGF19 expressions were significantly associated with phosphorylated AKT expression, suggesting that FGFR4 might be a therapeutic target for these patients (Jaakkola et al., 1993; Penault-Llorca et al., 1995; Tiong et al., 2016). In addition, FGFR4 overexpression is closely associated with advanced tumor stage and poor prognosis in astrocytomas. FGFR4 was also reported to be a prognostic marker in advanced stage, high grade serous ovarian cancer. Inhibiting FGFR4 and its ligand markedly reduces ovarian tumor growth both *in vitro* and *in vivo*, indicating that FGFR4 could also be a promising therapeutic target in ovarian cancer that has high expression of FGFR4 (Zaid et al., 2013). Similar results were also observed in prostate cancer. The expression of FGFR4 was markedly increased in prostate cancer and was associated with higher Gleason score and worse outcomes (Sahadevan et al., 2007; Sugiyama et al., 2010). FGFR4 transmembrane domain polymorphism (FGFR4 Gly388Arg) has been reported to be associated with increased risks of breast and prostate cancer in Asian population (Xu et al., 2010). FGFR4-Arg388 could activate ERK, c-scr and STAT3 signaling, contributing to tumor progression. Increased risk for developing the malignant tumor was reported in prostate, however, decreased risk was observed in head and neck squamous cell carcinoma (HNSCC) and soft tissue sarcomas for FGFR4-Arg388 allele. Accumulating evidences also suggested that FGFR4-Arg388 allele was frequently seen in rapid tumor progression, increased metastasis and advanced stage of patients with breast, prostate, colon and lung cancer (Heinzle et al., 2014).

Interaction between FGF19 and FGFR4 plays an essential role in colorectal tumorigenesis. Blocking either FGF19 or FGFR4 significantly reduces *in vivo* xenograft tumor growth of colon cancer cells (Pai et al., 2008). *In vitro*, knockdown of FGFR4 suppresses colon cancer cell proliferation and migration, again indicating that FGFR4 is a promising therapeutic target in colon cancer (Heinzle et al., 2012).

The main obstacle of targeted cancer therapy is the development of drug resistance. Significantly increased FGFR4 expression was observed in doxorubicin-resistant breast cancer cells, and knockdown of FGFR4 could increase sensitivity to doxorubicin. Mechanistically, expression of the anti-apoptotic protein BCL-XL was induced by increased FGFR4 through the MAPK signaling cascade (Roidl et al., 2009). Furthermore, activated FGF19-FGFR4 signaling has been reported to increase resistance against doxorubicin in basal-like breast cancer. Treatment of high-expressing FGF19 or FGFR4 positive breast cancer cells with an antibody against FGF19 or siRNA to FGF19 sensitizes the cells to doxorubicin (Tiong et al., 2016). Silencing FGFR4 sensitizes CRC cells to 5-fluorouracil (5-FU) or oxaliplatin treatment (Zhao H. et al., 2016).

The importance of FGF19-FGFR4 signaling is well-characterized in HCC. It has been reported that the activation of FGF19-FGFR4 signaling is important in multiple tyrosine kinase inhibitor (TKI), sorafenib resistance for the treatment of HCC. Overexpression of FGF19 could suppress sorafenib-induced, ROS-dependent apoptosis (Gao et al., 2017). Elevated FGF19-FGFR4 signaling is also able to reduce ROS-mediated apoptosis through inhibition of the caspase-3 pathway in colonic epithelial cells (Valastyan and Weinberg, 2011).

## Targeting the Fgf19-Fgfr4 Pathway

### FGFR4 Inhibitors

Currently, several FGFRs inhibitors are under investigation for the treatment of various cancers including breast, bladder, lung, liver, cholangiocarcinoma, and glioblastoma (Zaid et al., 2013). So far, some of these pan-FGFRs inhibitors have been reported to have efficient inhibitory effects for FGFRs including FIIN-2 (Masaru, 2016), JNJ-42756493 (Tabernero et al., 2015), LY2874455 (Tie et al., 2014), and ponatinib (Cortes et al., 2012). Infigratinib (BGJ398) selectively targets FGFR (1–3) with high affinity, but also targets FGFR4 with less affinity (Huynh et al., 2019). Meanwhile, accumulating evidence suggests that there could be more variability between FGFR4 and other FGFRs than was initially expected. It has been reported that there are a number of amino-acid substitutions in the tyrosine kinase domains of FGFR4 (Dutra et al., 2015; Katoh, 2016). FGF401 (Weiss et al., 2019; Zhou et al., 2019), BLU9931 (Hagel et al., 2015), and BLU-554 (Hatlen et al., 2019; Kim et al., 2019) have been reported to be the most highly potent and selective FGFR4 inhibitors. Representative FGFR4 inhibitors that are currently undergoing clinical trials were shown in Table 1. A phase I study of fisogatinib (BLU-554) validated that aberrant FGF19/FGFR4 signaling is a targetable oncogenic driver in HCC; fisogatinib was well-tolerated and most adverse events were manageable grade 1/2 gastrointestinal event – primarily diarrhea, nausea, and vomiting (Kim et al., 2019). Compared to the other three members of FGFRs, FGFR4 has one notably distinct amino acid change. Cys552 on FGFR4 is unique among the four paralogs of FGFRs and thus provides a selectivity handle to specifically target FGFR4. Cys552 in FGFR4 is required for the covalent binding with BLU9931 (Hoeflich et al., 2015). Selective FGFR4 targeting has been shown to reduce adverse effects, and the simultaneous targeting of FGFR4 and other receptor tyrosine kinases (RTKs) has been shown to indirectly enhance anti-tumor activity through normalization of the tumor microenvironment (Repana and Ross, 2015; Katoh, 2016).

**TABLE 1:** Overview of clinical trials involved in FGFR4 inhibitors.

**Drug**	**Clinical trial ID**	**Target(s)**	**Study design**	**Tumor types**	**Phase**	**Primary endpoint**	**Status**
Ponatinib	NCT02428543	Multipul RTKs, including Pan-FGFRs	40 participants Single group assignment Open label	Acute myeloid lukemia	I/II	Treatment	Recruiting

Erdafitinib JNJ -42756493	NCT03827850	Pan-FGFRs	50 participants Parallel assignment Open label	Squamous non-small cell lung carcinoma	II	ORR	Recruiting

FGF401	NCT02325739	FGFR4	172 participants Single group assignment Open label	HCC	I	Incidence rate of dose-limiting toxicity (DLT)	Completed

BLU-554	NCT02508467	FGFR4	150 participants Single group assignment Open label	HCC	I	Maximum tolerated dose (MTD) on qd and bid schedules	Recruiting

H3B6527	NCT0342457	FGFR4	17 participants Single group assignment Open label	Healthy participants	I	Mean area under the plasma-concentration time curve from time 0 through the last measurable concentration (AUC0-t) of H3B-6527	Completed

H3B6527	NCT02834780	FGFR4	128 participants Single group assignment Open label	Advanced hepatocellular carcinoma, intrahepatic cholangiocarcinoma, hepatocellular carcinoma	I	Number of participants with dose-limiting toxicities (DLTs) [time frame: escalation cycle 1 (21 days)]	Recruiting

INCB062079	NCT03144661	FGFR4	100 participants Parallel assignment Open label	Hepatocellular carcinoma (HCC), cholangiocarcinoma, esophageal cancer	I	Safety and tolerability of INCB062079 as measured by assessment of adverse events (AEs)	Recruiting

## Monoclonal Antibodies Against Fgf19 or Fgfr4

Another approach to targeting the FGF19-FGFR4 signaling pathway is the use of monoclonal antibodies against FGF19 or FGFR4. Desnoyers et al. (2008) produced a neutralizing anti-FGF19 monoclonal antibody that prevented liver cancer formation in FGF19 transgenic (FGF-TG) mice treated with diethylnitrosamine, a potent hepatocarcinogen which accelerate the development of HCC in FGF19-TG mice (French et al., 2012). The safety of this antibody was tested in cynomolgus monkeys, an appropriate model because there is similar binding affinity of anti-FGF19 to human and cynomolgus monkey FGF19. Treatment of monkeys with anti-FGF19 antibody increased bile acid synthesis and changed the expression of bile transporters in the liver (Pai et al., 2012). LD1 is a neutralizing antibody against FGFR4 that has been shown to inhibit FGFR4 mediated signaling, colony formation, and cell proliferation. LD1 is also able to suppress tumor growth in a preclinical model of liver cancer *in vivo* (Loilome et al., 2009). U3-1784, a novel FGFR4 targeting antibody, is a high-affinity fully humanized antibody. U3-1784 could compete with various FGFs for their FGFR4 binding sites, therefore inhibits receptor activation and downstream signaling, including FRS2 and ERK. Animal studies showed that U3-1784 exerts strong antitumor effects specific to liver cancer cells overexpressing FGF19 (Bartz et al., 2019). Strong antitumor effects and the lower toxicity of anti-FGFR4 antibody suggests a general strategy for avoiding adverse events with FGFR4 inhibitors for liver cancer therapy.

## Conclusion and Future Perspectives

Aberrant FGFR4 expression was observed in a number of human cancers, especially in HCC (Figure 1B) (Fischbach et al., 2015). Importantly, selective inhibitors of FGFR4 have demonstrated clinical benefit in HCC patients with high FGF19 expression (Kim et al., 2019). FGF19 binds to FGFR4 and activates GSK3β-Nrf2 signaling cascade to exert cytoprotective role against ER stress in HCC (Teng et al., 2017). It has been reported that both rearranged distal enhancement regions and point mutations in the proximal promoter region might cause FGFR4 overexpression (Koole et al., 2015). Moreover, inhibition of miRNAs targeting FGFR4 or aberrant long non-coding RNA (lncRNA) expression leads to FGFR4 overexpression (Masaru, 2016). FGFR4 inhibitors directly or indirectly suppress tumor growth by influencing the tumor microenvironment, particularly paracrine signaling, angiogenesis, and immune evasion (Masaru, 2016).

FGFR4 has been reported as an oncogenic driver in FGF19-positive advanced HCC. A therapeutic strategy against targeting FGF19/FGFR4 is being actively investigated (Dutra et al., 2015; Huynh et al., 2019). Meanwhile, inhibition of FGF19-FGFR4 signaling has been reported to be associated with increased risk of hepatotoxicity and cardiac toxicity (Lin and Desnoyers, 2012). Thus, careful preclinical studies evaluating the potential risks of treatment with FGFR4 inhibitors are critical to further development (Wang and Jacob, 2011; Foo et al., 2012).

Immune checkpoint blockade therapy has demonstrated several advantages in clinical oncology. PD-1 ligand (PD-L1) is presented in cancer cells and stromal/immune cells, while PD-1 and CTLA-4 are expressed in CD8^+^ T cells or Treg cells (Wu et al., 2014). Since both PD-1 and CTLA-4 signaling pathways are directly or indirectly involved in the inhibition of cytotoxic T cell functions; anti-PD-1, anti-PDL1, and anti-CTLA-4 monoclonal antibodies have been clinically used for cancer immunotherapy (Das et al., 2015). Interestingly, FGFR4 inhibitors have been shown to target immune cells in the tumor microenvironment, such as myeloid-derived suppressor cells (MDSCs) and M2-type tumor-associating macrophages (m2-tams), and are expected to indirectly inhibit PD-L1 expression in tumor cells and stromal/immune cells through influencing the tumor microenvironment (Masaru, 2016). Therefore, combination therapy with FGFR4 inhibitors and immune checkpoint blockers (anti-PD-1 or anti-CTLA-4 mAb) might be a promising therapeutic method for certain cancer patients.

In conclusion, the positive results of phase-I study for fisogatinib (BLU-554) in advanced HCC expressing FGF19 indicate that the development of covalently binding FGFR4 specific inhibitors is an important research direction. Meanwhile, searching for biomarkers that could predict the responses to FGFR4-targeting therapy are urgently needed. Another approach to target FGF19-FGFR4 signaling is monoclonal antibodies and FGF ligand traps which can block downstream signal of FGFR4 by interfering with ligand binding or receptor dimerization. Based on promising preliminary results from clinical trials of FGFR4 inhibitors, more efforts are needed to recognize patients who most likely benefit from treatment, and to design and implement effective combination therapies.

## Author Contributions

YL and MC collected the articles and made Figures. YC and CZ guided YL and MC to collect articles and make figures. XL prerevised the manuscript. CZ and RC wrote the manuscript and made a table.

## Conflict of Interest

The authors declare that the research was conducted in the absence of any commercial or financial relationships that could be construed as a potential conflict of interest.
